# Combining Injectable Plasma Scaffold with Mesenchymal Stem/Stromal Cells for Repairing Infarct Cavity after Ischemic Stroke

**DOI:** 10.14336/AD.2017.0305

**Published:** 2017-04-01

**Authors:** Hongxia Zhang, Fen Sun, Jixian Wang, Luokun Xie, Chenqi Yang, Mengxiong Pan, Bei Shao, Guo-Yuan Yang, Shao-Hua Yang, Qichuan ZhuGe, Kunlin Jin

**Affiliations:** ^1^Zhejiang Provincial Key Laboratory of Aging and Neurological Disorder Research, First Affiliated Hospital, Wenzhou Medical University, Wenzhou, China; ^2^Institute for Healthy Aging, University of North Texas Health Science Center at Fort Worth, TX 76107, USA; ^3^Department of Rehabilitation, Ruijin Hospital, School of Medicine, Shanghai Jiao Tong University, Shanghai, China; ^4^Med-x Research Institute, Shanghai Jiao Tong University, Shanghai, China

**Keywords:** senescence, aging, aging-related diseases, frailty, diagnosis, regulation

## Abstract

Stroke survivors are typically left with structural brain damage and associated functional impairment in the chronic phase of injury, for which few therapeutic options exist. We reported previously that transplantation of human embryonic stem cell (hESC)-derived neural stem cells together with Matrigel scaffolding into the brains of rats after focal ischemia reduced infarct volume and improved neurobehavioral performance. Matrigel is a gelatinous protein mixture extracted from mouse sarcoma cells, thus would not be approved for use as a scaffold clinically. In this study, we generated a gel-like scaffold from plasma that was controlled by changing the concentration of CaCl_2_. *In vitro* study confirmed that 10-20 mM CaCl_2_ and 10-40% plasma did not affect the viability and proliferation of human and rat bone marrow mesenchymal stem/stromal cells (BMSCs) and neural stem cells (NSCs). We transplanted plasma scaffold in combination of BMSCs into the cystic cavity after focal cerebral ischemia, and found that the atrophy volume was dramatically reduced and motor function was significantly improved in the group transplanted with scaffold/BMSCs compared with the groups treated with vehicle, scaffold or BMSCs only. Our data suggest that plasma-derived scaffold in combination of BMSCs is feasible for tissue engineering approach for the stroke treatment.

Stroke results in the disruption of cerebral tissue structures, and the neural cells begin to die within minutes. Persistent cell dysfunction and poor neural regenerative capabilities at the cerebral damaged site and beyond lead to the formation of irregular shaped lesions comprised of necrotic tissue and/or a fluid-filled cavity that are associated with prolonged neurological impairment. To date, no effective treatment is available for brain lesion in clinical settings. Current treatments, which have been focused on anti-inflammation and neuroprotection with pharmacological agents, have failed to produce clear improvements in the mortality and neurological outcome, perhaps due to their inability to structurally regenerate normal brain tissue at the lesion site [1].

Regenerative cell-based therapy offers long-term hope for many patients with stroke, as stem cells may render it possible for dead or injured neural cells to be replaced after acute stroke [2-4]. However, the efficiency and effectiveness are limited for chronic stroke patients due to brain cavity. Without the necessary biomechanical support and biochemical signals, neural stem cells (NSCs) may not have the ability to reconstitute lost neural tissue and to restore proper brain circuitry in the brain cavity [5, 6]. Regeneration of brain cavity in the chronic phase after stroke may become possible through a tissue engineering approach. Biodegradable scaffolds, which act as templates for tissue regeneration to guide the growth of new tissue[7], have been used for reconstruction after brain trauma [8-10], or spinal cord injuries [11, 12], but less attention has been given to their role in brain ischemia. Cerebral ischemia may be a particularly suitable target for such combined cell and scaffold treatment, since all cell types in the ischemic zone are affected and tissue architecture is significantly disrupted. This means that, in contrast to disorders that result in a selective degeneration of a single cell type, ischemia may require not only replacement of cells, but also restoration of a panoply of extracellularly derived chemotactic and haptotactic influences. A much more encouraging finding is that the structure of neonatal mouse brain injured by hypoxia-ischemia can be restored after transplantation of a polymer scaffold (synthetic) seeded with clonal murine neural progenitor cells [13]. In addition, Dr. Park *et al* implanted “biobridges” composed of NSCs seeded upon a polymer scaffolds into the evolving infarct cavity after ischemic stroke and found robust reciprocal interactions between exogenous implant and injured host brain ensued spontaneously, resulting in a substantial reduction in parenchymal loss and the reconstitution of anatomical connections [13]. To further help elucidate the potential of neural stem cell replacement therapy in stroke, we transplanted human embryonic stem cells (ESC)-derived NSCs into the infarct cavity after focal ischemia in rats. We found that transplantation of human ESC-derived NSCs with Matrigel scaffolding (natural) resulted in improved histologic and behavioral outcome [14]. However, the scaffold used in our previous work is the Matrigel®, a gelatinous protein mixture extracted from EHS mouse sarcoma cells. Therefore, there is almost no chance that this mouse sarcoma derived gelatin would be approved for use as a scaffold for grafting cells for humans.

In general, scaffolds can be classified into natural and synthetic categories. Natural materials including extracellular matrix (ECM) protein and their derivation (e.g., Laminin, collagen, fibronectin and gelatin) have features compatible and suitable for cell adherence, but these materials do not possess sufficient mechanical strength and degrade rather rapidly in the body if not chemically-cross linked [15]. Synthetic materials are attractive for their unique physical and chemical properties in terms of the controllable degradation rate, porosity, and mechanical strength [7]. However, they may not interact with cells in a useful way, as can biological derived materials, which may provide less than ideal for actual application in clinical setting. Importantly, any scaffold, whether natural or synthetic, may induce adverse immune or inflammatory reactions in the host.

To address this issue, we generated gel-like scaffold from plasma after adding blood coagulation substances, and then transplanted plasma-derived scaffold in combination of mesenchymal stem/stromal cells (BMSCs) into the cystic cavity after occlusion of the middle cerebral artery (MCAO). We found that the infarct lesion was dramatically reduced and motor function was significantly improved after transplantation. As the ideal scaffold should be nontoxic, biocompatible, and biodegradable, the autologous plasma-derived scaffold seems to be one suitable biomaterial, because of its availability and compatibility. Importantly, the plasma-derived scaffold can be generated from patient’s autologous blood, and the potential risk of allo- or xenograft-induced immune reactions or the transfection of pathogens is avoided and thus more clinically useful. In addition, using autologous plasma-derived scaffold may recapitulate the *in vivo* milieu and allowing cells to influence their own microenvironments [16, 17].

## MATERIALS AND METHODS

### Plasma scaffold preparation

The rats were anesthetized, and blood (3ml) was collected, via cannulation of the jugular vein, by adapting the technique of Harms and Ojeda (1974) [18]. The canula placed in the jugular vein was removed and the tissues was repositioned and sutured. In addition, the fresh plasma was also obtained from 5 healthy volunteer donors. The blood samples was transferred into commercially available anticoagulant-treated tubes (EDTA-treated tubes) and centrifuged at 700 *g* for 10 min in a cooling centrifuge at 4°C [19]. After centrifugation, the blood was separated in several layers. The top layer of the separation was aspirated and used for plasma-clot preparation. The platelet-containing plasma was transferred in Eppendorf microtubes and kept at -20°C. The plasma was mixed with different concentrations of blood coagulation substances, including fibrinogen (0-80mg), thrombin (0-250 IU/ml) and/or CaCl_2_ (0-100 mM; Dade Berhing, Marburg, Germany), for 60 min. The development of gel turbidity with time was monitored at 600nm with a spectrophotometer.

### BMSC isolation and culture

Human BMSCs (hBMSCs; n=8) were obtained from healthy volunteers at the First Affiliated Hospital of Wenzhou Medical University after informed consent, and all human experiments were approved by the ethics committee at the First Affiliated Hospital. The mean age of the healthy volunteers was 36 ± 5 years (ranging from 31 to 53 years). The bone marrow aspirate (3 ml) was diluted in PBS at a 1 to 1 ratio, and gently layered onto a Percoll cushion for density-gradient centrifugation. The low density of hBMSCs-enriched mononuclear cells was collected and re-suspended in α-MEM containing 10% FBS and plated at a concentration of 1X10^6^ cells/ml into a 25-cm^2^ tissue culture flask as previously described [20]. The cultures were then incubated at 37°C in a humidified atmosphere supplemented with 5% CO_2_. After 72 hrs, non-adherent cells in the supernatant were removed and the medium was completely replaced with fresh medium. hBMSCs (passage 3 to 5) were immunophenotypically characterized by flow cytometry using the following monoclonal antibodies (all from BD Biosciences): CD90-FITC, CD44-PE, CD73-APC, CD105-Percp 5.5, CD34-PE, CD11b-PE, CD19-PE, CD45-PE, and HLA-DR-PE.

Rat BMSCs were isolated and cultured as described previously [21]. In brief, the bone marrow was obtained from the femurs and tibias taken from male Sprague-Dawley (SD) rats. Harvested cells were sieved through a 70-μm nylon mesh and washed twice with medium, the BMC suspension was diluted with PBS and transferred slowly onto Percoll-Paque Plus, and the material was centrifuged for 25 min. The cells were then suspended in 5 ml DMEM-LG with 10% FBS (HyClone), 100 IU/ml of penicillin, 100 IU/ml of streptomycin (Sigma, St. Louis, MO) and plated in 25-cm^2^ flasks at a density of 1 X 10^6^ cells per dish. After 3 days of culture with 5% CO_2_ at 37°C, nonadherent hematopoietic cells were removed, and the medium was replaced. When the adherent cells grew to 80% confluence, samples were obtained and defined as passage zero (P0) cells. Rat BMSCs were immunophenotypically characterized by flow cytometry using the following monoclonal antibodies (all from eBioscience; 1:100 dilution): CD90-FITC, CD29-PE, CD45-APC.

### Neural stem/progenitor cell Culture

Neural stem/progenitor cells (NSCs) derived from human embryonic stem cell (*h*ESC) line BG01 were obtained from Aruna Biomedical Inc. (Athens, GA), and cultured as previously described [22]. Cells were seeded on polyornithine- and laminin-coated dishes and cultured in proliferation medium, consisting of Neurobasal medium with B27 supplementation containing 2 mM L-glutamine and 50 μg/ml Penn/Strep (all from Invitrogen, Carlsbad, CA), and 10 ng/ml leukemia inhibitory factor (LIF) plus 20 ng/ml fibroblast growth factor-2 (FGF-2) (both from R&D Systems; Minneapolis, MN) [23]. Cells were propagated further in proliferation medium and, upon reaching 90-100% confluence, were triturated to detach them from the dish.

### Cell viability assay

The effect of different plasma concentration on cell viability was assessed using the 3-(4,5-dimethylthiazohl-2-yl)-2,5-diphenyltetrazolium bromide (MTT; Sigma-Aldrich, USA) assay. Briefly, Immortalized mouse hippocampal cell line (HT-22 cells) were cultured using a standard protocol and seeded in 96-well plates. After treatment with different concentrations of rat plasma or 10% FBS for 24?hrs, the cells were incubated with MTT solution (Sigma-Aldrich) at 37?°C for 4?hrs. Then, the medium was removed, and 100?μl of dimethyl sulfoxide (DMSO) was added to each well. The absorbance was measured at a wavelength of 590?nm.

### Cell proliferation assay

Cell proliferation was assessed with the cell counting kit-8 (CCK-8) assay (Tongren Shanghai Co., Shanghai, China). BMSCs were plated onto 96-well plates (5?X?10^3^ cells/well) with different concentrations of CaCl_2_ in a triplicate pattern. Cells treated with vehicle were used as controls. Assays were performed from 1 to 3 day after plating by the addition of 100 μl of fresh medium in 10?µl of the CCK-8 solution for another 2?hrs at 37?°C. The culture plates were then shaken for 10 min and the optical density (OD) values were read at 450 nm. The assay was repeated 3 times.

### Cell adhesion assays

BMSCs were grown in black-walled plates and treated with different concentrations CaCl_2_ for 1, 2 and 3 days. Cell adhesion assay was performed using calcein-AM kit (R&D Systems) according to the manufactory instruction. Fluorescence values were obtained using a 485-nm excitation filter and a 520-nm emission filter in a fluorescence multi-well plate reader (Molecular Devices, Sunnyvale, CA).

### Focal cerebral ischemia

All animal procedures were approved by Institutional Animal Care and Use Committee of University of North Texas Health Science Center, and conducted according to the National Institutes of Health (NIH) Guide for the Care and Use of Laboratory Animals, and every effort was made to minimize suffering and to reduce the number of animals used. Young adult male SD rats (~280 g) were purchased from Charles River (Wilmington, MA). Male rats were anesthetized with 4% isoflurane in 70% N_2_O/30% O_2_ using a mask. Permanent middle cerebral arterial occlusion (MCAO) was performed according to procedures described previously [14, 24-31]. Briefly, the left external carotid artery was surgically exposed and dissected, and a monofilament nylon suture (3-0, 19 mm-long) was inserted from the external carotid artery into the left internal carotid artery to occlude the origin of the left middle cerebral artery. The external carotid artery was then ligated and the wound closed. After surgery, the rats were placed in a cage under an infrared heating lamp until they recovered from anesthesia. Rectal temperature was maintained at 37.0 ± 0.5°C using a thermostat-controlled heating pad (Harvard Apparatus, Holliston, MA). Physiological variables will be measured just prior to ischemia, 10 min after the onset of ischemia and 10 min after the onset of reperfusion using an ISTAT portable clinical analyzer and EC8+ cartridge (Heska).

### *Transplantation* after experimental stroke

Cells and/or scaffold were transplanted 3 weeks after induction of focal ischemia. Rats (n=6-10 per group) were re-anesthetized with 4% isoflurane in 70% N_2_O/30% O_2_ and placed in stereotaxic frames with a rat head holder. Burr holes were drilled with a dental drill, which was irrigated continuously with saline at room temperature to prevent overheating of the underlying cortex. Cultured BMSCs described above were harvested and resuspended at 1X10^6^ cells/μl in artificial cerebral spinal fluid (aCSF). BMSCs/scaffold, BMSCs alone, scaffold alone or vehicle (aSCF) alone were injected directly into the infarct cavity via a 10-µl Hamilton syringe driven by a syringe pump. The injection volume for the cavity transplants was 15-μl, a volume that was determined from estimates of the cavity size at 3 weeks after focal ischemia. Injections were made into the center of the cavity (0.7 mm anterior to the bregma, 4.5 mm lateral to the midline, 2.5 mm deep beneath the dura). In addition, cells, scaffold, vehicle or cell/scaffold will also be injected in 3-μl increments over a period of 10 min in the sham-operated rats. Following the injection, the needle will be kept in place for 5 min before slowly removing it from the injection site. After injections were completed, bone wounds were closed with bone wax, anesthesia was discontinued, and animals were returned to their cages.

### Measurement of infarct cavity volume

For lesion volume measurement, animals were sacrificed 6 weeks after transplantation. Brains were removed, 40-µm coronal brain sections were stained with hematoxylin and eosin stain (H&E). Damaged cavity, left hemisphere area, and total brain area were measured by a blinded observer using the NIH Image program, and areas were multiplied by the distance between sections to obtain the respective volumes. Volume of the lesion cavity was calculated as a percentage of the volume of the contralateral hemisphere, as previously described [32].

#### Behavioral testing

For functional assessment, comprehensive analysis of long-term effects of BMSCs alone, BMSCs/scaffolds, scaffolds alone or vehicle alone on rat behavior was performed each week up to 4 weeks after transplantation. The experimenters conducting behavioral testing and neurobehavioral scoring were blind to the experimental conditions. Each experimental group comprised of 10-11 male rats.

##### Bederson's test

Bederson’s test score was used to assess the neurological deficit using a four-level scale [33]: 0, normal; 1, forelimb flexion; 2, decreased resistance to lateral push; 3, circling.

##### Limb-placing test

Limb-placing test that assess the sensorimotor integration of the forelimb and the hind limb by checking responses to tactile and proprioceptive stimulation was performed according to the method as described by De Ryck et al [34] and modified by Puurunen K et al [35]. Both sides of the body were tested 24 hr after focal ischemia. The test consisted of seven limb-placing tasks, which were scored by a tester blind to the treatment groups. The following scores were used to detect impairment of the hindlimb and the forelimb: 0, no placing; 1, incomplete or delayed placing; 2, complete, immediate placing. Average placing scores (forelimb and hindlimb combined) for each week of testing were calculated for each animal.

##### Elevated Body Swing Test (EBST)

The elevated body swing test was used to test asymmetric motor behavior (8). Rats (n=6-8 per group) held by the base of the tail were raised ≈10 cm above the testing surface. The initial direction of body swing, constituting a turn of the upper body of >10° to either side, was recorded in three sets of 10 trials, performed over 5 min. The number of turns in each (left or right) direction was recorded, and the percentage of turns made to the side contralateral to the ischemic hemisphere (percent left-biased swing) was calculated. For each rat, average scores were determined.

##### Cylinder Test

The cylinder test, developed by Schallert *et al* [36], was used as a measure of forelimb asymmetry by observing the rat’s movements over 3-minute intervals in a transparent, 18-cm-wide, 30-cm-high transparent Plexiglas cylinder, which was sufficiently large to permit movement but small enough to promote rearing and wall exploration. A mirror behind the cylinder made it possible to observe and record forelimb movements when the rat was facing away from the examiner. After an episode of rearing and wall exploration, a landing was scored for the first limb to contact the ground or for both limbs if they made simultaneous contact. Percent use scores were calculated for both the unimpaired and impaired limb, relative to the total number of movements. Percentage use of the impaired limb was subtracted from percentage use of the unimpaired limb to yield an overall limb bias score. Wall exploration and landing movements were analyzed separately. Animals were tested 1-4 weeks after focal ischemia following transplantation.

##### Ladder rung walking test

Ladder rung walking test is sensitive for quantifying skilled locomotor movements. The degree of motor dysfunction after stroke is measured by counting the number of foot-faults of impaired limbs per round. The apparatus was modified to 122-cm-long horizontal plastic-rung runway at varying intervals of 7-14 mm between the rungs [37]. All animals will conduct 3 trials prior to perform the baseline testing. Baseline and post-operative testing trials consisted of 3 sessions. All kind of foot slip or total miss step will represent an error. The number of errors and steps by the affected forelimb and hindlimb in each trial will be counted. The mean errors in 3 sessions will be calculated.

**Figure 1.**
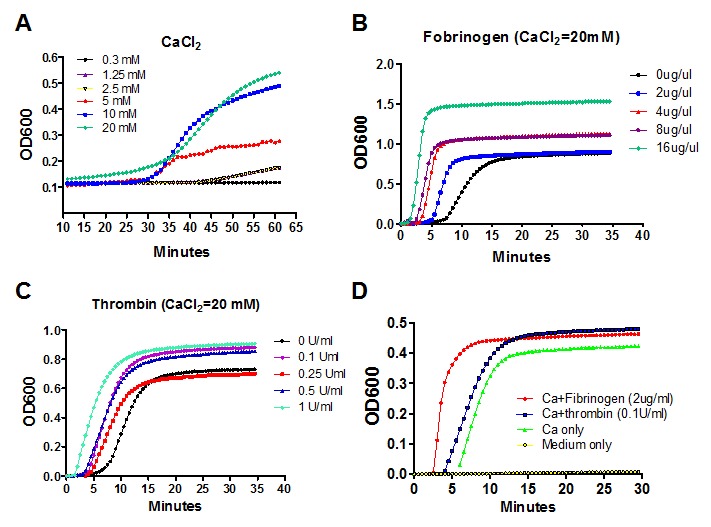
**The effect of CaCl_2_, fibrinogen and thrombin on the formation of plasma gel *in vitro***. A) The time course of development of gelatinous clot of plasma in the presence of different concentrations of CaCl_2_. B) The effect of different concentrations of fibrinogen on the formation of gelatinous clot of plasma. C) The effect of different concentrations of thrombin with 20 mM CaCl_2_ on the formation of gelatinous clot of plasma. D) The comparison of different combinations of coagulation factors on formation of gelatinous clot of plasma. The experiments were repeated three times.

### Statistical Analyses

Quantitative data were expressed as mean ± SEM from 3 - 4 experiments. Behavioral data were analyzed by two-way analyses of variance (ANOVA) with repeated measurements, followed by *post-hoc* multiple comparison tests (Fisher PLSD or Student’s paired *t* test with the Bonferroni correction). Damaged volume was analyzed by one-way ANOVA followed by Fisher PLSD *post-hoc* tests. *p* values <0.05 were considered significant.

**Figure 2.**
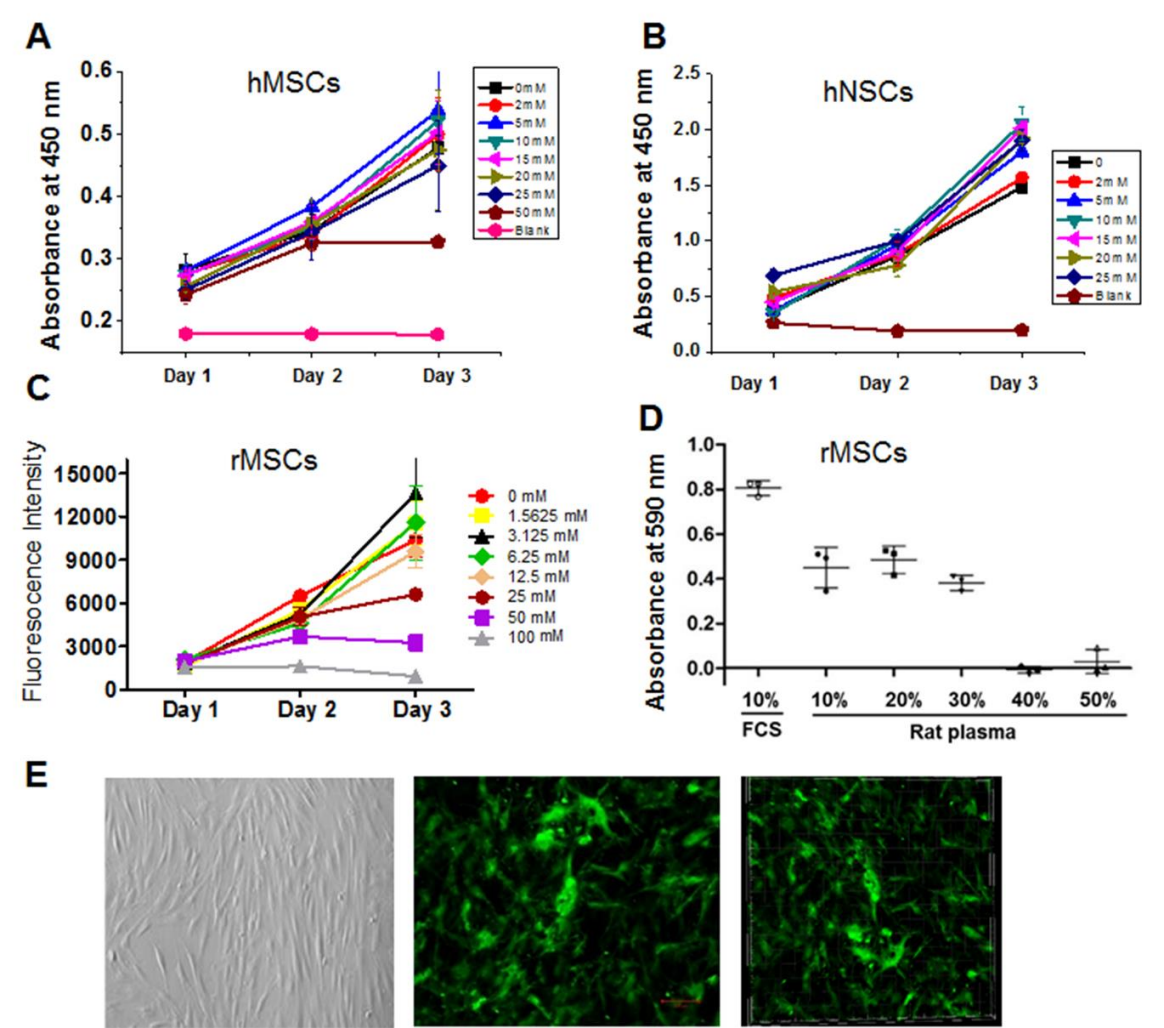
**The effect of different concentrations of CaCl_2_ and plasma on cell survival and death *in vitro***. A) The human BMSCs (hBMSCs) were treated with different concentrations of CaCl_2_ for 1, 2 and 3 days and CCK-8 proliferation assay was performed to determine the survival rate of hBMSCs. B) Human embryonic stem cells-derived NSCs were incubated with CaCl_2_ at different concentration as indicated and the proliferation rate of NSCs was determined by CCK-8 proliferation assay. C) Quantitation of calcein-AM fluorescent staining of rat BMSCs (rBMSCs) treated with different concentrations of CaCl_2_ for 1, 2 and 3 days. D) The HT-22 cells were treated with different concentrations of rat plasma and 10% FBS for 24 hrs and the survival rate of HT-22 cells was determined by MTT assay. E) The representative images of rBMSCs cultured in gelatinous plasma clot (30% rat plasma and 10uM CaCl_2_). Left panel: bright view of rBMSCs; middle panel: 2D view of GFP-positive rBMSCs; right panel: 3D view of GFP-positive rBMSCs. The experiments were repeated three times.

## RESULTS

Calcium ions (CaCl_2_) are required for the process of blood clotting. Addition of EDTA or citrate prevents clotting by binding calcium. Therefore, recalcified plasma can clot *in vitro* later by adding back an excess of calcium ions. As shown in Fig. 1A, recalcified plasma clotting occurred in the presence of CaCl_2_. The different concentration of CaCl_2_) not only affected the clotting time, but also increased the clot turbidity. In the presence of 20 mM CaCl_2_, the initial clotting time of recalcified plasma was shortened and the clot became more gelatinous by adding fibrinogen (Fig. 1B) or thrombin (Fig. 1C). In addition, the clot turbidity was increased, along with increased concentrations of fibrinogen or thrombin, and reached the platform after 15 min (Fig. 1B and C). The initial clotting time of recalcified plasma is slightly prolonger and the clot turbidity is little reduced in the group treated with CaCl_2_ compared with the group treated with either CaCl_2_/fibrinogen or CaCl_2_/thrombin (Fig. 1D).

**Figure 3.**
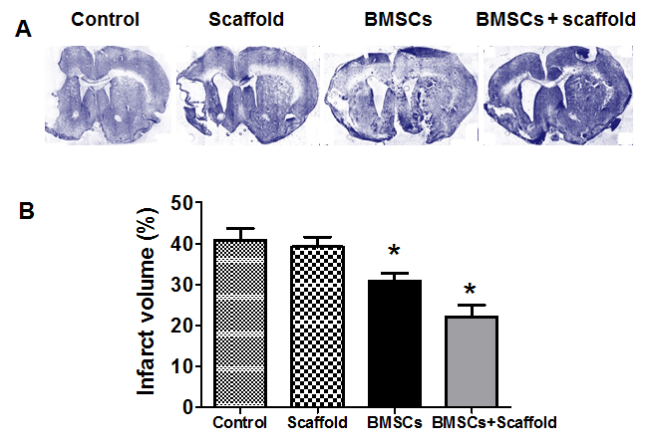
**BMSCs/plasma-derived scaffold transplantation reduced damaged volume of young adult rats after MCAO**. A) Representative images of lesion areas in H&E-stained stained coronal brain sections from scaffold and BMSCs/scaffold-treated rats at 6 weeks after dMCAO. B) Quantitative analysis of damaged volume in vehicle (control), scaffold, BMSCs and BMSCs/scaffold-treated young rats 4 weeks after transplantation.

Next, we tested whether CaCl_2_ at the different concentration affected the viability of human and rat BMSCs and NSCs *in vitro*. The hBMSCs were cultured in the presence of different concentrations of CaCl_2_ for 1, 2 and 3 days, and CCK-8 proliferation assay was then used to determine the proliferative rate of hBMSCs. As shown in Fig. 2A, CaCl_2_ at the concentrations of 2-25 mM did not affect the proliferation of hBMSCs. However, 50mM CaCl_2_ significantly reduced the proliferative rate of hBMSC at 3 days after treatment. Similarly, 2-25 mM CaCl_2_ did not affect the viability of human embryonic stem cells-derived NSCs based on CCK-8 proliferation assay (Fig. 2B). We also used calcein-AM fluorescent staining for assessment of rBMSCs treated with different concentrations of CaCl_2_ for 1, 2 and 3 days. We found that CaCl_2_ at the concentration of 50 and 100 mM reduced the viability of rBMSCs, but not at the concentration of less than 25 mM (Fig. 2C). In addition, we found that cell viability was over 95% in the presence of 10-30% plasma. However, the cell death determined by MTT assay was significantly increased when the cells were incubated with 40-50% plasma (Fig. 2D). We found that after 1 weeks under culture conditions, GFP-positive hBMSCs remained viable and proliferated with clot gelled in the presence of 30% human plasma and 10 mM CaCl_2_ (Fig. 2E).

Then, we determined if the transplantation of plasma-derived scaffold and BMSCs improved the outcome after ischemic stroke. We used a rat model of MCAO, which has become the model of choice for approximating the pathology of human stroke [38]. As expected, we found that the core of the infarct changes to a cystic cavity involving motor cortex and somatosensory cortex of both the forelimb and hindlimb regions was formed in 2-3 weeks after stroke, we thus performed experiment 3 weeks after focal ischemia. Rat BMSCs mixed with plasma-derived scaffolding, were transplanted 3 weeks post-MCAO into the infarct cavity of 3-mo-old SD rats. We found that infarct volume was reduced - by ~47% (*p* < 0.05) in the group implanted with scaffold and BMSCs and ~30% (*p* < 0.05) in the group implanted with BMSCs, compared with the group treated with vehicle (Fig. 3).

**Figure 4.**
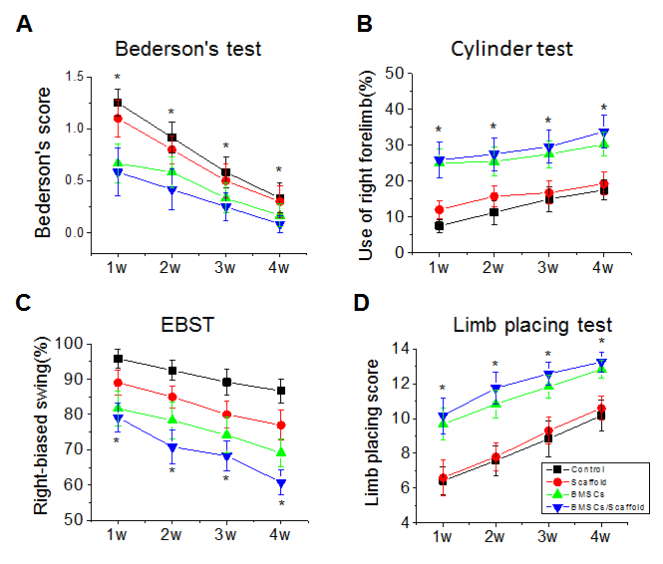
**BMSCs/plasma-derived scaffold transplantation improves long-term recovery after experimental stroke in young rats**. BMSCs, scaffold, vehicle (control) or BMSCs/scaffold was implanted into brain cavity 3 weeks after MCAO and neurological behavioral tests including Bederson's test (A), Cylinder Test (B), EBST (C), and limb placing test (D), were performed at 1-4 weeks after transplantation. Values presented as mean ± SEM. *P < 0.05, compared with vehicle-treated group.

Finally, we asked whether these plasma-derived scaffold and BMSC transplantation significantly contributed to long-term functional recovery after focal ischemia. Several behavioral tests have been developed that allow for the evaluation of more complex sensorimotor functions, such as EBST for basic postural reflexes and asymmetrical trunk function, and the cylinder test to detect asymmetry in forelimb use and sensorimotor integration. The ladder rung walking test is a new task to assess skilled walking and measure both forelimb and hindlimb placing, stepping, and inter-limb co-ordination [39]. Before MCAO, the Bederson test scores were similar among the groups, with most scores being a scale of zero, indicating no deficit (data not shown). Significant difference in Bederson scores was found before and after MCAO. Bederson neurological function was significantly improved in rats treated with scaffold and BMSCs or BMSCs only, compared with the rats transplanted with vehicle or scaffold (Fig. 4A). In the cylinder test, ischemic rats showed preferential use of the unaffected limb. The preference observed at 1-4 weeks was significantly reduced in rats transplanted with plasma-derived scaffold and BMSCs as well as BMSCs (Fig. 4B). In the EBST, rats showed a strong and persistent tendency to turn their upper bodies to the side opposite the ischemic hemisphere. The turning bias was significantly reduced after transplantation of scaffold and BMSCs or BMSCs, compared with vehicle-treated rats. Similarly, scaffold and BMSC-transplanted rats showed better performance in hindlimb placing test compared with the control group (Fig. 4D; *p*<0.05), and the effect persisted for up to 4 weeks. In addition, the scaffold and BMSC-transplanted group showed better results than the BMSC-transplanted group. In ladder rung walking test, the ischemic rats in four groups made a similar number of errors with their affected forelimbs when crossing a horizontal ladder one week after stroke. The scaffold and BMSC- and BMSC-transplanted group progressively recovered compared to the vehicle-treated group (Fig. 4E). However, the scaffold and BMSC-transplanted group showed better improved locomotor function than the BMSC-transplanted group.

**Figure 5.**
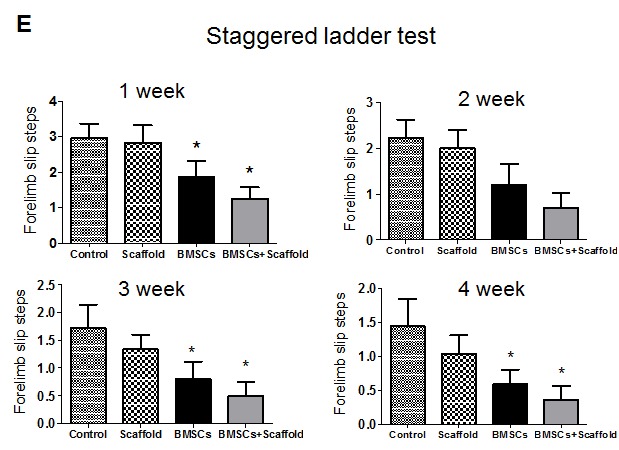
**BMSCs/plasma-derived scaffold transplantation improves long-term recovery after experimental stroke in young rats**. BMSCs, scaffold, vehicle (control) or BMSCs/scaffold was implanted into brain cavity 3 weeks after MCAO and staggered ladder test was performed at 1-4 weeks after transplantation. Values presented as mean ± SEM. *P < 0.05, compared with vehicle-treated group

## DISCUSSION

We found previously that transplantation of hESC-derived NSCs, together with Matrigel scaffolding, into young-adult SD rat brain 3 weeks after MCAO reduced infarct volume and improved neurobehavioral deficits [14]. In this study, we extended our study and found that clot formation could be controlled by changing the concentration of CaCl_2_ added to plasma. The plasma-derived scaffold did not impact the viability and proliferation of BMSCs and NSCs in vitro. In addition, we also found that the infarct lesion was reduced and motor function was improved after transplantation of rat BMSCs mixed with the plasma-derived scaffold into the brain cavity at 3 weeks after focal ischemia in young adult rats. Our data suggest that plasma-derived scaffold in combination of BMSCs is feasible for tissue engineering approach for stroke treatment, which makes it possible using patient’s autologous plasma as scaffold and autologous BMSCS for clinical applications.

Scaffolds is not only as a space filler, but also provide cells with a microenvironment necessary for tissue repair and regeneration and biochemical signals. In addition, scaffolds can provide a desirable way to restore tissue structure and function by recruiting tissue in-growth from the host surroundings [6].

We used plasma rather than serum for scaffold generation, as its rapid processing time, and containing fibrinogen and other clotting factors. In addition, plasma has a higher viscosity and total protein content than serum [40]. The gelation times and network architecture can be controlled by varying the calcium concentration, and form a wide array of soft substrates under physiological conditions, consisten with previous report [41]. The plasma-derived gel can be used as an injectable biodegradable scaffold, and allow them to deform to large cavity and stiffen but not break. Previous studies have documented that autologous plasma-derived clot is used as a biological scaffold for mesenchymal stem cells in treatment of orthopedic healing [17] and nerve repair [42]. As described above, natural or synthetic scaffold may induce adverse immune or inflammatory reactions in the host. Using plasma-derived scaffold can be generated from patient’s autologous blood without the potential risk of foreign body reaction or infection and is thus potentially clinically useful [16, 17].

One of biggest issues in cell replacement strategies is implant rejection in response to proteins from the donor cells that differ from those found in the recipient, although the brain is recognized as “an immunologically privileged site” [43]. BMSCs may be used as ideal cells for transplantation, since BMSCs have been therapeutically evaluated in animal models of stroke with substantial functional benefit, which have led to the initiation of a number of clinical trials worldwide in neural repair. BMSCs have also have other advantages, including that it is easy to obtain and expand these cells in culture, that using the patient's own BMSCs would eliminate the risk of rejection, and that BMSCs have the capacity to migrate to the injury site, permitting systemic administration [44].

The focal ischemia model we used in these experiments involved the occlusion of the left MCA proximal to the origin of the lateral lenticulostriate arteries, producing a reliably large cortical infarction that affects sensorimotor cortex including the rostral forelimb and caudal forelimb regions and extends to the lateral part of the striatum as well. As expected, we found that focal ischemia caused marked impairment in sensorimotor function and cognitive learning performance using comprehensive behavioral test batteries, which were sensitive enough to detect the effects of therapeutic agents and track behavioral recovery over time [38, 45]. A more significant improved outcome in behavioral tests were observed in the BMSCs and scaffold-implanted group, compared with implantation of vehicle in 1-4 weeks after transplantation. The mechanism through which BMSCs and scaffold transplantation reduces infarct size and improves functional outcome after focal ischemia is elusive. We did not find that implanted BMSCs could transdifferentiate into neural cells 2 months after transplantation by analysis of double immune-cytochemistry (data not shown). Therefore, behavioral improvement in animals after stroke may result from endogenous NSCs changes in response to transplants, which may be directly affected by growth factors released from implanted BMSCs. Recent studies show that transplanted cells also secrete trophic factors that help to maintain marginally surviving cells or otherwise enhance the local environment sufficiently to improve function. For example, a recent study shows that after subacute transplantation into the ischemic brain, the stem cell-secreted factor, human VEGF, is necessary for cell-induced functional recovery [46]. As it may take at least 1 month for NSC differentiation into mature neurons, early improvement of neurobehavioral outcome supports the notion that the neuronal differentiation may not be the major effect for functional outcome after NSCs.

The significance of the proposed work lies in the prospect that *in situ* brain tissue engineering using a combination of BMSCs and biodegradable plasma-derived scaffold might provide a basis for therapeutic interventions to improve the functional outcome in patients with disability after strokes. The goals of our studies are to help stroke survivors become as independent as possible and to attain the best possible quality of life. To reach the goals, the tasks ahead are challenging: what are the molecular and cellular mechanisms underlying involvement of histological and behavioral outcome mediated tissue engineering after stroke, which should be addressed in near further.
